# Lipid Mediators in Critically Ill Patients: A Step Towards Precision Medicine

**DOI:** 10.3389/fimmu.2020.599853

**Published:** 2020-11-25

**Authors:** Luca Cioccari, Nora Luethi, Mojgan Masoodi

**Affiliations:** ^1^Department of Intensive Care Medicine, Inselspital, Bern University Hospital, Bern, Switzerland; ^2^Australian and New Zealand Intensive Care Research Centre, School of Public Health and Preventive Medicine, Monash University, Prahran, VIC, Australia; ^3^Department of Emergency Medicine, Inselspital, Bern University Hospital, Bern, Switzerland; ^4^Institute of Clinical Chemistry, Inselspital, Bern University Hospital, Bern, Switzerland

**Keywords:** critical illness, inflammation, resolution of inflammation, lipidomics, fatty acid-derived lipid mediators

## Abstract

A dysregulated response to systemic inflammation is a common pathophysiological feature of most conditions encountered in the intensive care unit (ICU). Recent evidence indicates that a dysregulated inflammatory response is involved in the pathogenesis of various ICU-related disorders associated with high mortality, including sepsis, acute respiratory distress syndrome, cerebral and myocardial ischemia, and acute kidney injury. Moreover, persistent or non-resolving inflammation may lead to the syndrome of persistent critical illness, characterized by acquired immunosuppression, catabolism and poor long-term functional outcomes. Despite decades of research, management of many disorders in the ICU is mostly supportive, and current therapeutic strategies often do not take into account the heterogeneity of the patient population, underlying chronic conditions, nor the individual state of the immune response. Fatty acid-derived lipid mediators are recognized as key players in the generation and resolution of inflammation, and their signature provides specific information on patients’ inflammatory status and immune response. Lipidomics is increasingly recognized as a powerful tool to assess lipid metabolism and the interaction between metabolic changes and the immune system *via* profiling lipid mediators in clinical studies. Within the concept of precision medicine, understanding and characterizing the individual immune response may allow for better stratification of critically ill patients as well as identification of diagnostic and prognostic biomarkers. In this review, we provide an overview of the role of fatty acid-derived lipid mediators as endogenous regulators of the inflammatory, anti-inflammatory and pro-resolving response and future directions for use of clinical lipidomics to identify lipid mediators as diagnostic and prognostic markers in critical illness.

## Introduction

Systemic inflammation is a common pathophysiological feature of many conditions encountered in the intensive care unit (ICU). A key determinant of the outcome in critically ill patients is the balance of pro- vs. anti-inflammatory pathways and the body’s capability to resolve the acute inflammation and restore homeostasis. An appropriate and timely inflammatory response protects the body from the injurious agent and eliminates the threat without causing collateral damage. However, a dysregulated inflammatory response can contribute to multiple organ dysfunction and early in-hospital death (1, 2).

Fatty acid-derived lipid mediators play a pivotal role in the endogenous regulation of infection and inflammation (3, 4). In recent years, the resolution of inflammation and restoration of homeostasis has been recognized as an active process. Specialized pro-resolving mediators (SPMs) derived from polyunsaturated fatty acids (PUFA) have been detected as key signaling molecules in the resolution of inflammation and play an important role in dampening the inflammatory response without causing immunosuppression (5, 6).

The human immune response is complex, highly variable and unpredictable, and ICU patients represent an exceptionally heterogeneous population. There is a growing recognition that treating ICU patients requires a more personalized approach. Precision medicine offers a strategy for prevention and treatment of disease based on characteristics of each individual to maximize effectiveness, and, therefore, can overcome some challenges associated to ICU patients (7–11). In addition to genetics and clinical data often used in precision medicine (12), assessing metabolism using metabolomics and lipidomics can provide valuable information for further phenotyping and characterization of patients. Lipidomics provides a powerful tool to assess lipid metabolism and identify specific lipid profiles in such patients (3, 13–15), thus providing unique insights into the individual immune response. Identification of such metabolic signatures could improve prognostic and diagnostic evaluation and pave the path to personalized treatment strategies.

In this review, we address the role of fatty acids-derived bioactive lipid mediators and their prognostic, diagnostic and therapeutic potential in frequently encountered intensive-care related conditions.

## Fatty Acid-Derived Lipid Mediators: Endogenous Regulators of Inflammation and Resolution

**Figure 1:**
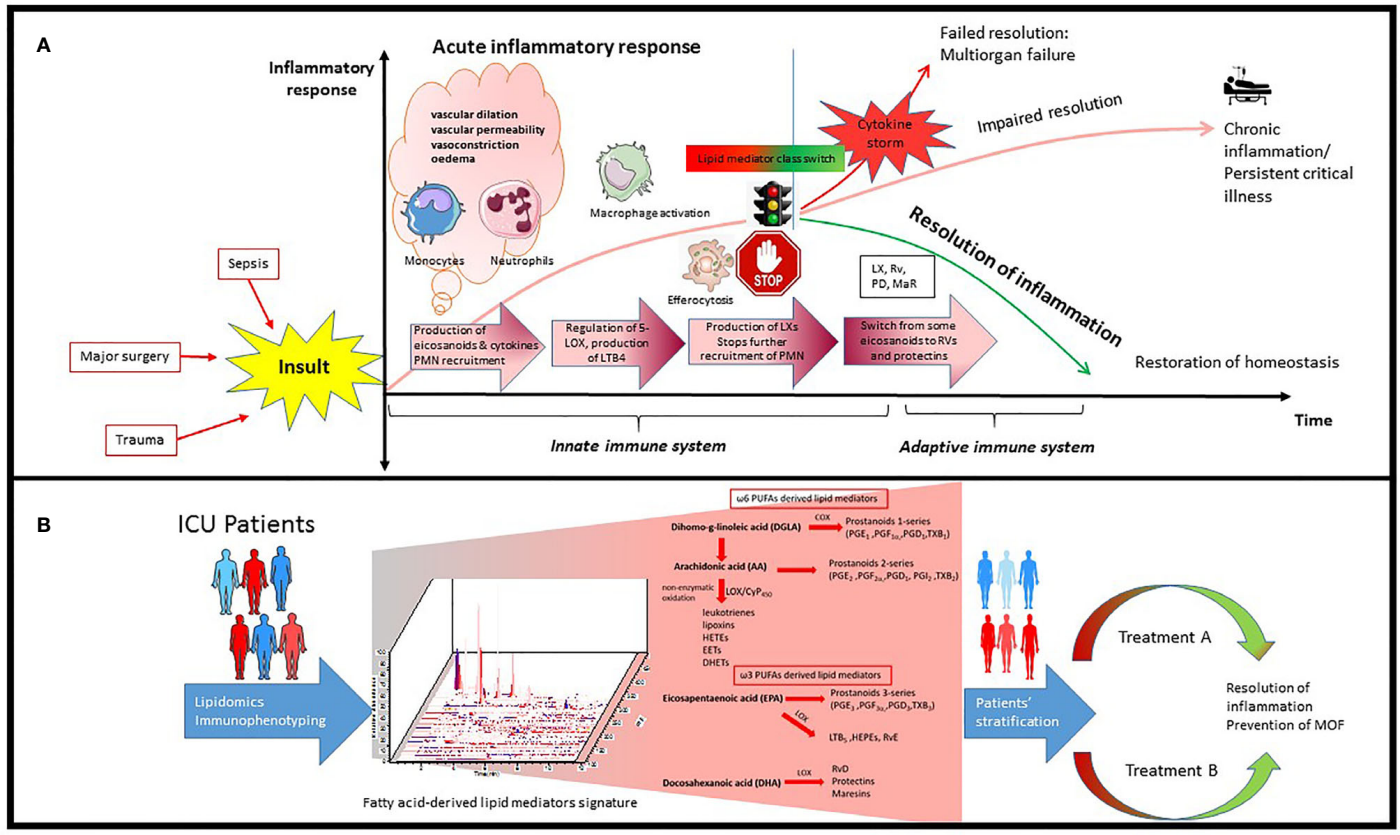
Schematic process of inflammatory response in ICU patients and how it can be used for precision medicine. **(A)** Inflammatory insults like bacterial infection and trauma leads to rapid influx of immune cells, mainly neutrophils and monocytes, followed by monocyte infiltration and differentiation to inflammatory macrophages. This process is orchestrated by pro-inflammatory lipid mediators such as eicosanoids and cytokines. Resolution of inflammation is highly dependent on the signaling network generated during this process as well as alteration in number and phenotype of macrophages and lymphocytes. PGE2 can also activate the regulation of 15-LOX in human neutrophils, which leads to production of lipoxins and stops further recruitment of PMN. There is an active switch from production of some eicosanoids to resolvins and protectins which initiates the resolution of inflammation. **(B)** Lipidomics provides a powerful tool to identify and quantify hundreds of fatty acid-derived lipid mediators simultaneously potentially participating and contributing to inflammation and its resolution which leads to identification of specific signatures in ICU patients. Integrating transcriptomics, proteomics and lipidomics could further advance our understanding of this complex network during infection in ICU patients, leading to better patient stratification and personalized treatment.

In the normal immune response, the acute inflammation is followed by successful resolution and repair of tissue damage. However, upon dysregulation of the immune response, persistence of inflammation leads to immune suppression and organ failure (16, 17). Inflammatory insults such as tissue damage or microbial invasion activate cells of the innate immune system like macrophages and dendritic cells to initiate a nonspecific immune response (18) which leads to rapid influx of immune cells, mainly neutrophils and monocytes, followed by monocyte differentiation into inflammatory macrophages. This process is orchestrated by pro-inflammatory lipid mediators such as eicosanoids (e.g., prostaglandins and leukotrienes), cytokines (e.g., TNF, IL-1, IL-6), and chemokines (19) (**Figure 1A**). Prostaglandins are produced by most cells in our body and act as autocrine and paracrine lipid mediators upon stimulation (e.g., mechanical trauma, growth factor, cytokines), while leukotrienes are produced predominantly by inflammatory cells like macrophages, polymorphonuclear leukocytes, and mast cells (20). Pro-inflammatory prostaglandins like prostaglandin E2 (PGE2) and leukotriene B4 (LTB4) initiate and contribute to the characteristic inflammatory response which includes vascular dilation, vascular permeability and edema (21, 22).

Resolution of inflammation is highly dependent on the signaling network generated during this process as well as alterations in number of lymphocytes and phenotype of macrophages (23). The acute inflammatory response is normally terminated once the triggering insult is eliminated. However, when excess neutrophils congregate, they can cause additional tissue damage, and sometimes lead to unresolved chronic inflammation (3, 24–26) (**Figure 1A**).

In recent years, the resolution of inflammation and restoration of homeostasis have been recognized as active processes, regulated by a superfamily of endogenous lipid mediators, namely specialized proresolving mediators (SPMs). SPMs include ω-6 arachidonic acid-derived lipoxins, ω-3 eicosapentaenoic acid (EPA) and docosahexaenoic acid (DHA)-derived resolvins, protectins and maresins (4, 27, 28) (**Figure 1B**). These novel immunoresolvents are key signaling molecules in the resolution of inflammation, enhancement of bacterial clearance, and play an important role in dampening the inflammatory response (29, 30).

PGE2 not only stimulates LTB4-mediated polymorphonuclear neutrophil (PMN) recruitment to sites of inflammation but also initiates resolution of inflammation by stimulating 15-lipoxygenase (LOX)-dependent lipoxin production in neutrophils (22, 31). Lipoxin then stimulates further production of other SPM (32), such as resolvins and protectins (33). Lipoxygenation and epoxidation of DHA lead to biosynthesis of maresins (*ma*crophage mediators in *re*solving *in*flammation) which, in turn, regulate the production of the leukocyte chemoattractant LTB_4_ (34). At the cellular level, lipoxins and resolvin E1 (RvE1) are potent stopping signals for further neutrophilic infiltration (35, 36). To remove the already infiltrated neutrophils from the tissue, Lipoxin A_4_ (LXA_4_) also stimulates macrophage efferocytosis (phagocytosis of apoptotic neutrophils and cell debris) (3). Epoxy lipid mediators generated *via* CYP450 have also been reported to limit the accumulation of inflammatory monocytes during resolution and exhibit a critical role in monocyte lineage recruitment and resolution (37).

Beyond innate phagocyte responses to resolve acute inflammation, SPMs appear to play critical roles in regulating adaptive immunity. SPMs selectively regulate cytokines *via* specific SPM receptors expressed on innate lymphoid, NK-, T-, and B cells (24).

SPM restrain inflammation and resolve infection, and each SPM family member possesses potent pro-resolving and anti-inflammatory actions [reviewed in (3)] with specific functions in the resolution phase (24). Several reports in experimental models demonstrated important roles for SPMs in promoting a return to homeostasis after infection or injury, leading to improved outcomes and survival (38). **Table S1** summarizes lipid mediators in animal models of intensive care-related conditions. Defects in SPM pathways impair the coordinated resolution of inflammation and could be implicated in the dysregulated inflammatory response encountered in many ICU-related conditions. Nevertheless, further and stronger evidence is needed to clarify the effects and potential role of SPMs in critical care.

## Lipid Mediators in Intensive Care-Related Conditions

### Sepsis

The hallmark of sepsis is a dysregulated host response to infection. Sepsis is defined as infection-related organ dysfunction, and septic shock is further complicated by refractory hypotension with elevated blood lactate levels (39–41). The pathophysiology of sepsis is extraordinarily complex (42). Various molecules originating from the infecting microorganism, so-called pathogen-associated molecular patterns (PAMPs), or from necrotic cells, the damage-associated molecular patterns (DAMPs) activate the innate immune system through pattern recognition receptors on leukocytes, leading to a signaling cascade eventually resulting in the generation of pro-inflammatory cytokines. This “cytokine storm” is likely responsible for the systemic inflammatory response and the resulting organ dysfunction (induced by both cellular infiltration and ischemia) characteristic of sepsis. As numerous attempts aiming to dampen this cytokine storm have failed in clinical trials (43), considerable challenges remain in the management of sepsis.

Administration of SPM has shown some promising results in animal models of sepsis; however, this approach has not yet been translated into clinical practice. In animal studies, administration of D-series resolvins counter-regulates proinflammatory genes, decreases excessive cytokine production, neutrophil recruitment and infiltration, and enhances phagocytosis of bacteria, reducing tissue damage and improving survival (44–51). Exogenous administration of maresins (52, 53) and lipoxins has similar effects (54–58).

Published human studies to date are mainly observational (**Table 1**). In addition, some clinical studies investigated aspirin-triggered resolvins and lipoxins. In healthy adults, low-dose aspirin stimulates biosynthesis of anti-inflammatory mediators (69) and, in ICU patients with a severe inflammatory response, it reduces the concentration of proinflammatory mediators (17-HETE, 18-HETE, and 20-HETE) and increases the concentration of the anti-inflammatory mediators 17,18-DiHETE and 14,15-DiHETE (60). However, Dalli et al. reported significantly higher levels of pro-resolving mediators like RvE1, RvD5 and 17R-PD1 in sepsis non-survivors compared to survivors (61). It is therefore arguable that higher levels of SPM might be harmful rather than useful. One possible explanation for this apparent contradiction is that, in sepsis non-survivors, the endogenous increase in SPM may not be sufficient to reverse the inflammatory process or perhaps the time window in which these mediators are produced is critical. Moreover, the increased levels of pro-inflammatory cytokines observed in non-survivors (61) suggest more severe systemic inflammation, where, although increased, SPM levels are not sufficient to resolve the ongoing inflammation. This hypothesis has also been supported by Abdoulnour and colleagues, who found that increased plasma 15-epi-LXA4 levels at baseline were associated with development of ARDS, indicating engagement of counter-regulatory pathways that were ultimately insufficient to prevent the development of ARDS in these patients (63). Finally, many SPM possess dual biological actions and their effect may change over time, as exemplified by the study of Sordi et al. (58): In mice, LXA4 was increased at the beginning of sepsis, contributing to the harmful excessive inflammatory response. However, LXA4 administered in *late* sepsis was beneficial to the animal, controlling the excessive inflammation. These data suggest that both antagonizing LXA4 actions in the beginning or its administration in later periods could be beneficial in sepsis treatment.

**Table 1:** Clinical lipidomics (or studies) of fatty acid–derived lipid mediators in intensive care–related conditions.

Setting	Mediator	Biological action/role	Reference
**Sepsis /SIRS**			
66 patients with sepsis 20 healthy controls	Lipoxin	– Baseline LXA_4_ levels were lower in sepsis patients (vs healthy controls) but not associated with 28-day mortality.	(59)
RCT of Aspirin (ASA) vs placebo 48 patients with SIRS (n=32 with lipid analyses)	Resolvins, Protectins, Maresins, Lipoxins	– ASA increased serum concentration of 15-HETE (LXA4 precursor) and anti-inflammatory mediators 17,18-DiHETE and 14,15-DiHETE. – ASA reduced the concentration of the proinflammatory mediators 17-HETE, 18-HETE, and 20-HETE.	(60)
22 patients with sepsis	Leukotriene Resolvins, Protectins PDX	– Higher 10*S*,17*S*-diHDHA (PDX) at day 3 predicted ARDS development. – Higher inflammation-initiating mediators (PGF2α, LTB4) and pro-resolving mediators (RvE1, RvD5, and 17R-PD1) in non-survivors.	(61)
**Acute lung injury/ARDS**			
Substudy of the LIPS-A trial (62), RCT of ASA vs placebo for prevention of ARDS: 345 patients at risk for ARDS	Thromboxane B2 (TXB2) Aspirin-triggered lipoxin A4 (ATL)	– ASA significantly decreased TXB2 and increased the plasma ATL/TXB2 ratio. – Elevated ATL associated with ARDS.	(63)
21 patients with ARDS	TXB2, prostaglandin F1-alpha (PGF1-alpha) and leukotriene B4 (LTB4)	– Plasma levels of eicosanoids higher in ARDS patients. – LTB4 correlated with the severity of respiratory failure.	(64)
16 patients with ARDS	TXB2, 6-keto prostaglandin F(1alpha), and LTB4	– LTB4 correlated with lung-injury severity and outcome.	(65)
**Traumatic brain injury (TBI)**			
15 patients with TBI 73 healthy controls	Free fatty acid (FFA) concentrations in cerebrospinal fluid (CSF)	– CSF concentration of all FFAs significantly higher in TBI patients. – Individual concentrations of arachidonic, myristic, and palmitic acids at 1 week significantly lower in patients with favorable early outcome compared to patients with worse outcome ratings at the time of hospital discharge.	(66)
**Trauma**			
100 trauma patients 20 healthy controls	Leukotriene B_4_	– Elevated LTB_4_-levels at admission predicted risk of pulmonary complications.	(67)
96 trauma patients 28 healthy controls	Lipid mediator gene pathways	– Higher resolvin pathway gene expression and lower gene expression ratio of leukotriene:resolvin pathways in patients with uncomplicated recovery.	(68)

ARDS, Acute Respiratory Distress Syndrome; ASA, acetylsalicylic acid; ATL, Aspirin-triggered lipoxin; CSF, cerebrospinal fluid; diHDHA, Dihydroxy-docosahexaenoic acid; DiHETE, Dihydroxy-eicosatetraenoic acid; FFA, free fatty acids; HETE, Hydroxyeicosatetraenoic acid; LT, Leukotriene; MaR, Maresin; PD, Protectin; PG, Prostaglandin; RCT, randomized controlled trial; Rv, Resolvin; SIRS, Systemic Inflammatory Response Syndrome; TBI, Traumatic brain injury; TX, Thromboxane.

### Acute Respiratory Distress Syndrome (ARDS)

Acute respiratory distress syndrome (ARDS) is characterized by a non-cardiogenic pulmonary edema (70), caused either by pulmonary or extrapulmonary events including severe pneumonia, sepsis, aspiration of gastric content, and trauma. The resulting acute lung injury is driven by excessive inflammation as a consequence of an imbalance of pro-inflammatory and anti-inflammatory cytokines, with release of multiple mediators of inflammation into the alveolar space and into the bloodstream (71). Increased endothelial and epithelial permeability then leads to alveolar fluid accumulation and impaired gas exchange. Resolution of ARDS requires endothelial and epithelial repair and reabsorption of alveolar edema fluid, and SPM are an essential component of the resolution program (72). Despite improvements in clinical management, mortality remains high and there is no specific treatment, nor are there universally agreed-upon biomarkers for survival and outcome in ARDS.

Different types of acute lung diseases have distinct lipid profiles (73) and lipid mediators may represent useful prognostic markers in critically ill patients. LTB4 correlates with lung-injury severity and outcome in patients with ARDS (64, 65) and higher pro-inflammatory mediators like PGF2α and selected pro-resolving mediators like 10S,17S-diHDHA were predictive of ARDS development in patients with sepsis (61). In patients at risk for ARDS randomized to aspirin versus placebo, increased levels of aspirin-triggered lipoxin A4 (15-epi-LXA4) were associated with the development of ARDS (63).

In animal models, administration of SPM has particularly beneficial effects in injured lungs (74). Maresins have organ protective effects, decrease edema, improve lung mechanics and tissue hypoxia (75). RvD1 decreases pulmonary edema, leukocyte infiltration and the release of pro-inflammatory cytokines and alleviates lung injury (76–79) and RvE1 can restore mitochondrial function in human alveolar epithelial cells and accelerates the resolution of experimental lung inflammation (80–82). Moreover, protectin D1 has beneficial effects in influenza-infected mice (83) and 15-epiLXA4 inhibits neutrophil infiltration and enhances pathogen clearance (84, 85).

### Trauma, Traumatic Brain and Spinal Cord Injury

Major trauma is a leading cause of morbidity and mortality around the globe (86, 87). Severe traumatic injury has a considerable impact on the immune and metabolic system (88, 89) and leads to a posttraumatic cascade of inflammatory changes (90–93). Therefore, lipid mediators have been proposed as prognostic markers in trauma patients (67, 68, 94). In patients with traumatic brain injury (TBI), cerebrospinal fluid concentration of free fatty acids is significantly elevated and correlates with clinical outcomes (66).

Accumulating evidence from animal studies suggests that various lipid mediators may have a role as therapeutic agents in cerebral and spinal cord injury. Elovanoids are derivatives from very long chain PUFAs and have neuroprotective properties in animal models of TBI and ischemic stroke (95, 96). In other animal models of TBI, RvD1 promotes functional recovery and halts glial activation and neuronal death, and RvE1 modulates the inflammatory response (97, 98). Moreover, parenteral or enteral administration of DHA reduces lesion size and axonal injury in rodents with TBI (99–101). The effect of DHA administration in rats with spinal cord injury has recently been summarized in a systematic review and meta-analysis (102). The reported studies suggest that, in rats, DHA can promote motor functional recovery after spinal cord injury. This effect appears limited to administration of DHA, and is not observed with EPA (103). Finally, Maresin 1 also improves neurological outcomes after experimental spinal cord injury (104). Although these findings are encouraging, further validation with adequate animal models are needed, taking into consideration the dose, target specificity and central nervous system penetration of tested compounds.

### Cerebral Ischemia and Reperfusion: Ischemic Stroke and Cardiac Arrest

Ischemia/reperfusion injury is a major determinant of poor outcome in patients with ischemic stroke and cardiac arrest survivors (105). In cardiac arrest, global cerebral ischemia alters cell metabolism and the balance of cerebral vasodilator/vasoconstrictor eicosanoids, rendering the cells susceptible to further damage after reperfusion: Vasoconstrictor eicosanoids are increased, and inhibition of 20-HETE synthesis (a potent vasoconstrictor) improves cortical perfusion and short-term neurologic outcome in a rat model of cardiac arrest (106).

In ischemic stroke, various *in vitro* and *in vivo* studies demonstrated that SPMs reduce leukocyte infiltration and neuronal injury, enhance efferocytosis and decrease both the production of inflammatory cytokines and oxidative stress (107). Cerebral artery occlusion and reperfusion causes significant reduction in endogenous RvD2 levels, and treatment with RvD2 reduces cerebral infarction, inflammatory cytokines, edema and neurological dysfunction (108). In another animal model, RvD1 promotes functional recovery, reduces neuroinflammation and prevents neuronal cell death (109). Neuroprotectin D1 (NPD1) down-regulates apoptosis and promotes cell survival (110, 111), and the administration of its precursor DHA has similar beneficial effects in experimental stroke (112–114). Additional administration of aspirin leads to cerebral synthesis of aspirin-triggered NPD1 (AT-NPD1), which reduces infarct size and significantly improves neurological scores in rats (110).

### Myocardial Infarction

As with stroke, ischemia/reperfusion plays a pivotal role in the pathophysiology of myocardial infarction and contributes to up to 50% of the final infarct size (115). A crucial aspect is the balance between vasoconstrictive and vasodilatory metabolites of arachidonic acid (116). Vasodilating epoxyeicosatrienoic acids (EETs) have cardioprotective effects (117, 118), and increasing EETs *via* administration of selective soluble epoxide hydrolase inhibitors shows beneficial effects in animal models of ischemia/reperfusion injury (119–122). Moreover, lipoxin administration post myocardial infarction improves left ventricular ejection fraction in mice (123). RvD1 promotes the resolution of acute inflammation initiated by myocardial infarction and has renoprotective effects, delaying the onset of heart failure and cardiorenal syndrome (124, 125). Finally, RvE1 prevents apoptosis in cardiac myocytes exposed to ischemia/reperfusion and decreases infarct size in rats (126). These experimental data suggest a potential for therapeutic use of SPMs in patients with myocardial infarction, however, no clinical studies have been published to date.

### Acute Kidney Injury (AKI)

Acute kidney injury (AKI), a frequent complication of critical illness, occurs in more than 50% of ICU patients (127). As management of AKI is largely supportive, early identification of patients at risk is of paramount importance. Several novel biomarkers for early detection of kidney damage have been identified (127), but limitations in specificity and sensitivity have prevented their clinical application. As early lipid changes are involved in the pathogenesis of AKI (128, 129), lipidomic analysis offers—once more—a promising approach for identifying diagnostic and prognostic biomarkers (130). Moreover, SPM have been studied as potential therapeutic agents in AKI due to their organ-protective properties in ischemia/reperfusion (131). In mice, administration of RvD or PD1 before an ischemic insult results in reduced functional and morphological kidney injury (132, 133) and aspirin-triggered resolvin D1 down-regulates the inflammatory response and protects against endotoxin-induced AKI (134).

In summary, analysis and characterization of specific lipid mediator profiles has the potential to improve diagnostic and prognostic accuracy in various conditions commonly encountered in the ICU. Numerous experimental studies provide a theoretical basis for therapeutic administration of lipid mediators in specific circumstances. However, translation from bench to bedside is still in its infancy.

## Conclusion and Future Directions

Systemic inflammation is a common pathophysiological trait of many conditions leading to critical illness. While a certain degree of inflammation is protective, a dysregulated inflammatory response is detrimental, contributing to multiple organ failure and death. Many clinical trials of treatments aiming at modulating the inflammatory response in ICU patients have failed to improve outcomes, partly due to the tremendous complexity and heterogeneity of critical illness. Hence, there is growing interest in personalized treatment in ICU patients (7–11). In past decades, the complexity of the human inflammatory response may have been under-recognized, and previous experimental and clinical models may not accurately represent human pathobiology (135–137). Lipidomics has attracted a lot of attention in recent years due to its ability to assess lipid metabolism and comprehensively characterize different molecular lipid species in different pathophysiological conditions. Recent advances in lipidomic research have highlighted the role of fatty acid-derived lipid mediators as key players in generation and resolution of inflammation. There are several challenges associated to profiling of such mediators, namely similar chemical structure with diverse biological functions as well as their low abundance in biological systems (13–15). This is further complicated by the dynamic biosynthesis of these molecular species that is time and cell-type dependent (4). Despite these challenges, several advancements related to the identification of novel mediators and the function of these mediators can be attributed to lipidomics approach, especially in animal models (138, 139). Computational and experimental models of bioactive lipid metabolism in human polymorphonuclear leukocytes has also been used to further assess the flux of these mediators in specific immune cells (140, 141). Although there have been several studies in animals, characterization of these lipid mediators in critical ill patients has not been established due to the additional complexity and heterogeneity of the patient population. Despite its complexity, lipidomics in critical illness has the potential not only to improve our understanding of the pathophysiological processes involved in generation and resolution of inflammation, but also to identify metabolic signatures or novel specific biomarkers for earlier diagnosis, better risk stratification and prediction of patient outcomes. Finally, it facilitates metabolic assessment providing valuable information for phenotyping and characterization of critically ill patients and may promote the steps towards precision medicine.

## Author Contributions

All authors contributed equally to the manuscript, writing sections of initial draft and then each revising other sections. Funding not applicable. All authors contributed to the article and approved the submitted version.

## Conflict of Interest

The authors declare that the research was conducted in the absence of any commercial or financial relationships that could be construed as a potential conflict of interest.
