# Supplementary material for: Lipid Mediators in Critically Ill Patients: A Step Towards Precision Medicine
Source: Front Immunol. 2020 Nov 25;11:599853. doi: 10.3389/fimmu.2020.599853 (PMC7724037; doi:10.3389/fimmu.2020.599853)
Supplement: Supplementary file 1 [file Table_1.pdf]

Table S1. Lipidomics of fatty acid-derived lipid mediators in animal models of intensive care-related conditions

Mediator	Model	Intervention	Biological action / role	Reference
**Sepsis**				
**Lipoxin**	Caecal ligation and puncture (CLP)-induced sepsis (P. aeruginosa) (mouse)	**LXA_4** (7 mg/kg i.v.) 1 h after surgery	- Increased neutrophil phagocytic ability and Fcg receptor I (CD64) expression.<br>- Decreased pathogen virulence.	(1)
	CLP-induced sepsis (rat)	**LXA_4** (40 µg/kg, i.p.) 5 h after surgery.	- Reduced blood bacterial load.<br>- Reduced systemic inflammation and NF-κB activation.<br>- Increased 8-day survival.	(2)
	E. coli peritonitis (mouse)	**15-epi- LXA_4** (1 µg/mouse) or placebo (saline) with antibiotics (ceftazidime)	- Attenuated systemic inflammation, inhibited bacteria dissemination.<br>- Reduced production of IL-6 and TNF-α by peritoneal macrophages.<br>- Increased survival.	(3)
	CLP-induced sepsis (mouse)	**LXA_4 compounds (at 7 µg/kg; i.v.)** (**LXA_4** compared to the stable 15-epi-16-(para-fluorophenoxy)-lipoxin A4)	- Both reduced plasma TNFα and IL-6 concentrations.<br>- reduced blood bacterial load with LXA4.<br>- LXA4 increased 8-day survival, the LXA4 analogue did not have a significant effect.	(4)
	Pneumosepsis (K. pneumoniae) (mouse)	**Lipoxin receptor agonists and antagonists** in early (1h) and late (24h) sepsis	- Treatment with receptor *antagonists* and inhibition of 5- and 15-lipoxygenase *1 h after infection,* improved leukocyte migration to the infected tissues and survival.<br>- Receptor *agonist and LXA4 worsened* early infection and reduced migration of leukocytes.<br>- 24 h after infection, LXA4 *improved* survival.	(5)
**Maresin**	CLP-induced sepsis (mouse)	**MaR1** (100ng i.p.)	- Reduced bacterial load and pro-inflammatory cytokines (TNF-α, IL-6).<br>- Increased survival rate.<br>- Attenuated liver & lung injury.	(6)
	CLP-induced sepsis (mouse)	**MaR1** (10, 50, and 100 ng) i.p.)	- Improved survival.<br>- Reduced bacterial growth, downregulated ROS production, decreased lactic acid, lowered TNF-α, IL-1β, MPO, MIP-2 and IL-10.	(7)

**Resolvin D**	*Citrobacter rodentium* intestinal infection and diarrhoea (mouse)	**RvD1 and RvD5** (100ng i.p.)	- Reduced bacterial load.<br>- Reduced inflammation.<br>- Increased survival.	(8)
	CLP-induced sepsis (mouse)	**RvD2** (100 ng) iv and/ or (1 μg) i.p. at 1h post-CLP	- RvD2 reduced cytokine levels.<br>- Enhanced bacterial clearance in blood and peritoneum at 12 h post-CLP.	(9)
	E. coli peritonitis (mouse)	**RvD1 and RvD5**	- RvD1, RvD5 reduced bacterial titres in blood and exudates and increased survival.<br>- RvD1, RvD5, and PD1 enhanced phagocytosis of *E. coli.*<br>- RvD5 counter-regulates pro-inflammatory genes, incl. NF-κB and TNF-α.<br>- SPM (RvD1, RvD5, PD1) plus ciprofloxacin heightened host antimicrobial response.	(10)
	Peritonitis (mouse)	Transfected Macrophages with or without **RvD2 (10 ng/mouse, i.p.)**	- RvD2 limited PMN infiltration and enhanced efferocytosis.	(11)
**ARDS/pneumonia**				
**Lipoxin**	E. coli pneumonia (mouse)	**15-epi-LXA$_4$** (100ng i.v.)	- Inhibition of lung neutrophil infiltration.<br>- Inhibition of NF-κB activation.<br>- RvD1 and RvD3 had an additive inhibitory effect with 15-epi-LXA4 on NF-κB phosphorylation.<br>- Enhanced pathogen clearance.	(12)
	Acute lung injury (mouse)	**15-epi-LXA$_4$** (200 μg/kg) i.v. after 24h of injury	- Accelerated resolution of neutrophil-dependent pulmonary inflammation.	(13)
**Maresin**	ARDS (mouse)	**MaR1** (10 ng i.v., ~0.5 μg/kg) 1 h after HCl	- Organ-protective effects: decreased lung neutrophils, oedema, tissue hypoxia, and pro-inflammatory mediators.<br>- Improved lung tissue hypoxia and lung mechanics.	(14)
**Protectin, Resolvin, Lipoxin**	Severe influenza by H5N1 (mouse)	**PUFA-derived** lipids mediators (**12-HETE, 15-HETE, 17-HDoHE, and PD1**) (1 μg/mouse) or vehicle (i.v.) 12 hr before and immediately after virus infection	- Improved survival.<br>- PD1 markedly attenuated virus replication.<br>- RvD1, RvD2, or LXA4 treatment did not decrease the proinflammatory cytokine levels (IL-6, IP-10) in the lungs.	(15)

**Resolvin D**	Mechanical ventilation-induced lung injury (mouse)	**RvD1** (500 ng i.p.)	- Reduction in lung damage, pulmonary oedema, leucocyte infiltration and TNF-α, IL-1β and IL-6.	(16)
	Chemical alveolar epithelial injury (mouse)	**RvD1**	- Attenuation of lung fibrosis and reduction of mechanical stretch-induced mesenchymal markers.	(17)
	Hydrochloric acid-induced acute lung injury (mouse)	**AT- RvD1** (100ng)	- Reduces lung resistance.<br>- Enhances restitution of barrier function.	(18)
	LPS-induced acute lung injury (mouse)	**RvD1** (1 µg/kg or 5 µg/kg) 30 min before inducing ALI by LPS	- Improved pathological lung changes.<br>- Reduced leukocytes recruitment, TNF-α and IL-6 production in bronchoalveolar lavage fluids<br>- Improved survival.	(19)
	Acute lung injury (mouse)	**AT-RvD3** (10 ng/mouse) i.v. 1h after injury	- Reduced histopathological signs of acid injury (alveolar wall thickening, oedema, and leukocyte infiltration).	(20)
**Resolvin E**	Aspiration pneumonia (mouse)	**RvE1** (~0.005 mg/kg) i.v. prior to acid injury	- Decreased lung neutrophil accumulation<br>- Enhanced clearance of E. coli.<br>- Decreased lung tissue levels of proinflammatory chemokines and cytokines (incl. IL-1β, IL-6)<br>- Improved survival.	(21)
	Neutrophil-mediated acute lung injury (mouse)	**RvE1** (25 µg/kg, i.p.) three times at 4-h intervals).	- Enhanced resolution of pneumonia, attenuated lung inflammation.<br>- Improved survival.	(22)
**Spinal cord injury**				
**DHA, EPA**	Compression spinal cord injury (rat)	**DHA or EPA** (both 250 nmol/kg) or saline i.v. 30 min. after insult	- DHA significantly reduced number of neutrophils in areas of injury at 4 h and 24 h.<br>- DHA reduced c-reactive protein (CRP) plasma levels.<br>- EPA did not reduce neutrophils or CRP.	(23)
**Maresin**	Compression spinal cord injury (mouse)	**1 µg of MaR1 i.v.** (repeated daily until d7)	- Increased resolution at lesion site and improved neurological outcome.	(24)
**Traumatic brain injury (TBI)**				
**DHA**	Controlled cortical impact injury (mouse)	**DHA (500 nmol/kg)** or saline i.v. 30 min post-injury.	- DHA reduced neurological deficit and increased pro-resolving mediators in the injured brain.<br>- DHA reduced lesion size, axonal injury at 7 days post-TBI.	(25)

			- DHA reduced neurofilament light chains levels in plasma at 28 days.	
	Controlled cortical impact injury (CCI) (rat)	**DHA** i.p. 30 min after cortical impact	- DHA blunted increased mRNA levels of pro-inflammatory genes at day 3 and 7 and of anti-inflammatory genes at day 30.<br>- DHA improved performance on memory test at day 14 after CCI.	(26)
	Impact acceleration injury (rat)	**DHA** either 10 mg/kg/d or 40 mg/kg/d enteral supplementation for 30 days	- Reduced Amyloid-Precursor-Protein proteins in axons at 30 days post-injury, to levels similar to seen those in uninjured animals.	(27)
**Resolvin D and E**	Midline fluid percussion injury (mouse)	**RvE1 (100ng daily), AT-RvD1 (100ng daily)** daily for 7d beginning 3days prior to TBI.	- RvE1 treatment modulated post-traumatic sleep and the inflammatory response to TBI.<br>- Improvement in motor and cognitive outcome seen in AT-RvD1-treated mice.	(28)
**Ischemia-reperfusion**				
**20-HETE**	Asphyxic cardiac arrest (rat)	inhibiting the synthesis of vasoconstrictor **20-HETE**	- Accelerated neurologic recovery, reduced cortical neurodegeneration.<br>- Decreased cerebral water content after cardiac arrest.	(29)
**Resolvin**	Ischemia-reperfusion-induced **second-organ injury** (mouse)	**Resolvins and analogues** at 1 µg/mouse (i.e., DHA, RvD1, RvE1)	- RvD1, but not RvE1or DHA, protects lung tissues from excessive leukocyte infiltration.	(30)
**Stroke**				
**Resolvin D**	Cerebral ischemia-reperfusion by middle cerebral artery occlusion (rat)	**RvD2** (25, 50, and 100 µg/kg)	- Attenuation of IL-6 and TNF-α at infarct site.<br>- Reduced infarct area and neurological dysfunction.	(31)
	Focal brain injury, hemicerebellectomy (rat)	**RvD1** 0.4 µg/kg, i.p. on days 0, 3, 5, and 7	- Promotion of functional recovery.<br>- Reduced neuroinflammation.<br>- Increased neuronal survival.	(32)
**DHA**	Middle cerebral artery occlusion (rat)	**DHA (i.v.) (Neuroprotectin D1)** 1 h after onset of reperfusion	- Improves neurological scores, protects ischemic penumbra, decreases MRI lesions and diminishes infarct volumes.	(33)
**Acute myocardial infarction (MI)**				

**EET**	Left coronary artery occlusion and 2h reperfusion (rat)	**8,9-EET** (2.5 mg/kg), **11,12-EET** (2.5 mg/kg) or **14,15-EET** (2.5 mg/kg) i.v.	- 11,12-EET and 14,15-EET significantly reduced myocardial infarct size in rats as compared with control.	(34)
	Left coronary artery occlusion and 2h reperfusion (mouse)	**sEH inhibitor and/or 14,15- EET**, i.v. 15 min before LCA occlusion or during ischemia 5 min before reperfusion.	- Increasing 14,15-EET was cardioprotective against ischemia-reperfusion injury.	(35)
**Lipoxin**	Permanent coronary ligation (mouse)	**Lipo-15-epi-LXA_4** or **15-epi-LXA_4** (1 µg/kg/day) was injected 3 hours post-MI for (d)1 or continued daily until d5.	- Improved left ventricular (LV) ejection fraction at day 5.	(36)
**Resolvin D**	Permanent coronary ligation (mouse)	**RvD1** (3ug/kg/d; s.c., 3h after MI)	- Increased neutrophil clearance from infarcted LV, and reparative macrophages at day 5.<br>- Attenuated MI-induced renal inflammation, decreasing neutrophil gelatinase-associated lipocalin and proinflammatory cytokines and chemokines.	(37)
	Permanent coronary ligation (mouse)	**RvD1** (3µg/kg/day) injected 3 hr post-myocardial infarction (MI) for day (d)1 until d 5	- Improved LV function (fractional shortening).<br>- Decreased collagen deposition and reduced post-MI fibrosis.<br>- RvD1 and Lipo-RvD1 promoted the resolution of acute inflammation initiated by MI.<br>- Increased levels of pro-resolving mediators RvD1, RvD2, Maresin 1 (MaR1) and Lipoxin A4 (LXA4) in spleens from RvD1 injected mice at day 5 post-MI.	(38)
**Resolvin E**	Left coronary artery occlusion and 2h reperfusion (rat)	**RvE1** (0, 0.03, 0.1, or 0.3mg/kg) iv before reperfusion	- RvE1 dose-dependently reduced infarct size.	(39)
**Acute kidney injury (AKI)**				
**Resolvin D**	LPS-induced AKI (mouse)	**AT-RvD1**<br>(1 µg/mouse) i.p. 1h after LPS	- AT-RvD1 protects against LPS-induced AKI.<br>- AT-RvD1 diminished LPS-induced renal tubular damage.	(40)
	LPS-induced AKI (mouse)	**RvD1 (**5 µg/kg)	- Decreased TNF-α level, ameliorated kidney pathological injury, protected kidney function.<br>- Improved survival.<br>- Down-regulation of NF-κB inflammatory signal and inhibition of renal cell apoptosis.	(41)

**Resolvin D, Protectin (PD1)**	Bilateral ischemia/reperfusion kidney injury (mouse)	**RvDs** (10 ug/mouse), **RvD1** (10 ug/mouse), **PD1** (10 ug/mouse), or vehicle.	- RvDs or PD1 markedly attenuate ischemic kidney injury as well as reduce fibrosis.<br>- RvDs and PD1 reduce number of infiltrating leukocytes and blocked toll-like receptor (TLR)-mediated activation of macrophages.	(42)

Abbreviations: AKI; acute kidney injury. ARDS; Acute Respiratory Distress Syndrome. ASA; acetylsalicylic acid. AT-RvD; Aspirin-triggered Resolvin D. ATL; Aspirin-triggered lipoxin. CCI; Controlled cortical impact. CLP; Caecal ligation and puncture. CRP; C-reactive protein. CSF; cerebrospinal fluid. DHA; Docosahexaenoic acid. diHDHA; Dihydroxy-docosahexaenoic acid. DiHETE; Dihydroxy-eicosatetraenoic acid. EET; Epoxyeicosatrienoic acid. EPA; Eicosapentaenoic acid. FFA; free fatty acids. HETE; Hydroxyeicosatetraenoic acid. IL-6; Interleukin-6. IP-10; Interferon gamma-induced protein 10. i.p.; intra-peritoneal. LV; left ventricle. Lipo-15-epi-LXA4; liposomal 15-epi-LXA4. LPS; Lipopolysaccharide. LT; Leukotriene. MaR; Maresin. MI; myocardial infarction. NF-κB; nuclear factor kappa-light-chain-enhancer of activated B cells. PD; Protectin. PG; Prostaglandin. PUFA; Polyunsaturated fatty acids. RCT; randomized controlled trial. Rv; Resolvin. sEH; Soluble Epoxide Hydrolase. SIRS; Systemic Inflammatory Response Syndrome. SPM; Specialized pro-resolving mediators. TBI; Traumatic brain injury. TNF-α; Tumour necrosis factor α. TX; Thromboxane.

Bibliography

1. Wu B, Capilato J, Pham MP, Walker J, Spur B, Rodriguez A, et al. Lipoxin A4 augments host defense in sepsis and reduces Pseudomonas aeruginosa virulence through quorum sensing inhibition. *The FASEB Journal* 2016; 30(6):2400-2410. 10.1096/fj.201500029R.
2. Walker J, Dichter E, Lacorte G, Kerner D, Spur B, Rodriguez A, et al. Lipoxin a4 increases survival by decreasing systemic inflammation and bacterial load in sepsis. *Shock* 2011; 36(4):410-416. 10.1097/SHK.0b013e31822798c1.
3. Ueda T, Fukunaga K, Seki H, Miyata J, Arita M, Miyasho T, et al. Combination therapy of 15-epi-lipoxin A4 with antibiotics protects mice from Escherichia coli-induced sepsis*. *Crit Care Med* 2014; 42(4):e288-295. 10.1097/CCM.0000000000000162.
4. Wu B, Walker Ja Fau - Temmermand D, Temmermand D Fau - Mian K, Mian K Fau - Spur B, Spur B Fau - Rodriguez A, Rodriguez A Fau - Stein TP, et al. Lipoxin A(4) promotes more complete inflammation resolution in sepsis compared to stable lipoxin A(4) analog. (1532-2823 (Electronic)).
5. Sordi R, Menezes-de-Lima O, Jr., Horewicz V, Scheschowitsch K, Santos LF, Assreuy J. Dual role of lipoxin A4 in pneumosepsis pathogenesis. *Int Immunopharmacol* 2013; 17(2):283-292. 10.1016/j.intimp.2013.06.010.
6. Hao Y, Zheng H, Wang R-H, Li H, Yang L-L, Bhandari S, et al. Maresin1 Alleviates Metabolic Dysfunction in Septic Mice: A ¹H NMR-Based Metabolomics Analysis. *Mediators of Inflammation* 2019; 2019:2309175. 10.1155/2019/2309175.
7. Gu J, Luo L, Wang Q, Yan S, Lin J, Li D, et al. Maresin 1 attenuates mitochondrial dysfunction through the ALX/cAMP/ROS pathway in the cecal ligation and puncture mouse model and sepsis patients. (1530-0307 (Electronic)).
8. Diaz LA, Altman NH, Khan W, Serhan CN, Adkins B. Specialized proresolving mediators rescue infant mice from lethal Citrobacter rodentium infection and promote immunity against reinfection. *Infection and Immunity* 2017; 85(10). 10.1128/IAI.00464-17.
9. Spite M, Norling LV, Summers L, Yang R, Cooper D, Petasis NA, et al. Resolvin D2 is a potent regulator of leukocytes and controls microbial sepsis. *Nature* 2009; 461(7268):1287-1291. 10.1038/nature08541.
10. Chiang N, Fredman G, Backhed F, Oh SF, Vickery T, Schmidt BA, et al. Infection regulates pro-resolving mediators that lower antibiotic requirements. *Nature* 2012; 484(7395):524-528. 10.1038/nature11042.
11. Chiang N, Dalli J, Colas RA, Serhan CN. Identification of resolvin D2 receptor mediating resolution of infections and organ protection. *J Exp Med* 2015; 212(8):1203-1217. 10.1084/jem.20150225.
12. Sham HP, Walker KH, Abdulnour R-EE, Krishnamoorthy N, Douda DN, Norris PC, et al. 15-epi-Lipoxin A₄, Resolvin D2, and Resolvin D3 Induce NF-κB Regulators in Bacterial Pneumonia. *The Journal of Immunology* 2018; 200(8):2757. 10.4049/jimmunol.1602090.
13. El Kebir D, Jozsef L, Pan W, Wang L, Petasis NA, Serhan CN, et al. 15-epi-lipoxin A4 inhibits myeloperoxidase signaling and enhances resolution of acute lung injury. *Am J Respir Crit Care Med* 2009; 180(4):311-319. 10.1164/rccm.200810-1601OC.

14. Abdulnour RE, Dalli J, Colby JK, Krishnamoorthy N, Timmons JY, Tan SH, et al. Maresin 1 biosynthesis during platelet-neutrophil interactions is organ-protective. *Proc Natl Acad Sci U S A* 2014; 111(46):16526-16531. 10.1073/pnas.1407123111.
15. Morita M, Kuba K, Ichikawa A, Nakayama M, Katahira J, Iwamoto R, et al. The lipid mediator protectin D1 inhibits influenza virus replication and improves severe influenza. *Cell* 2013; 153(1):112-125. 10.1016/j.cell.2013.02.027.
16. Xia H, Wang J, Sun S, Wang F, Yang Y, Chen L, et al. Resolvin D1 Alleviates Ventilator-Induced Lung Injury in Mice by Activating PPARγ/NF-κB Signaling Pathway. (2314-6141 (Electronic)).
17. Yang Y, Hu L, Xia H, Chen L, Cui S, Wang Y, et al. Resolvin D1 attenuates mechanical stretch-induced pulmonary fibrosis via epithelial-mesenchymal transition. *Am J Physiol Lung Cell Mol Physiol* 2019; 316(6):L1013-l1024. 10.1152/ajplung.00415.2018.
18. Eickmeier O, Seki H, Haworth O, Hilberath JN, Gao F, Uddin M, et al. Aspirin-triggered resolvin D1 reduces mucosal inflammation and promotes resolution in a murine model of acute lung injury. *Mucosal Immunol* 2013; 6(2):256-266. 10.1038/mi.2012.66.
19. Wang B, Gong X, Wan JY, Zhang L, Zhang Z, Li HZ, et al. Resolvin D1 protects mice from LPS-induced acute lung injury. *Pulm Pharmacol Ther* 2011; 24(4):434-441. 10.1016/j.pupt.2011.04.001.
20. Colby JK, Abdulnour RE, Sham HP, Dalli J, Colas RA, Winkler JW, et al. Resolvin D3 and Aspirin-Triggered Resolvin D3 Are Protective for Injured Epithelia. *Am J Pathol* 2016; 186(7):1801-1813. 10.1016/j.ajpath.2016.03.011.
21. Seki H, Fukunaga K, Arita M, Arai H, Nakanishi H, Taguchi R, et al. The anti-inflammatory and proresolving mediator resolvin E1 protects mice from bacterial pneumonia and acute lung injury. *Journal of immunology (Baltimore, Md : 1950)* 2010; 184(2):836-843. 10.4049/jimmunol.0901809.
22. El Kebir D, Gjorstrup P, Filep JG. Resolvin E1 promotes phagocytosis-induced neutrophil apoptosis and accelerates resolution of pulmonary inflammation. *Proc Natl Acad Sci U S A* 2012; 109(37):14983-14988. 10.1073/pnas.1206641109.
23. Hall JC, Priestley Jv Fau - Perry VH, Perry Vh Fau - Michael-Titus AT, Michael-Titus AT. Docosahexaenoic acid, but not eicosapentaenoic acid, reduces the early inflammatory response following compression spinal cord injury in the rat. (1471-4159 (Electronic)).
24. Francos-Quijorna I, Santos-Nogueira E, Gronert K, Sullivan AB, Kopp MA, Brommer B, et al. Maresin 1 Promotes Inflammatory Resolution, Neuroprotection, and Functional Neurological Recovery After Spinal Cord Injury. *J Neurosci* 2017; 37(48):11731-11743. 10.1523/jneurosci.1395-17.2017.
25. Thau-Zuchman O, Ingram R, Harvey GG, Cooke T, Palmas F, Pallier PN, et al. A Single Injection of Docosahexaenoic Acid Induces a Pro-Resolving Lipid Mediator Profile in the Injured Tissue and a Long-Lasting Reduction in Neurological Deficit after Traumatic Brain Injury in Mice. *J Neurotrauma* 2020; 37(1):66-79. 10.1089/neu.2019.6420.
26. Schober ME, Requena DF, Casper TC, Velhorst AK, Lolofie A, McFarlane KE, et al. Docosahexaenoic acid decreased neuroinflammation in rat pups after controlled cortical impact. (1090-2430 (Electronic)).

27. Bailes JE, Mills JD. Docosahexaenoic acid reduces traumatic axonal injury in a rodent head injury model. *J Neurotrauma* 2010; 27(9):1617-1624. 10.1089/neu.2009.1239.
28. Harrison JL, Rowe RK, Ellis TW, Yee NS, O'Hara BF, Adelson PD, et al. Resolvins AT-D1 and E1 differentially impact functional outcome, post-traumatic sleep, and microglial activation following diffuse brain injury in the mouse. *Brain Behav Immun* 2015; 47(1090-2139 (Electronic)):131-140. 10.1016/j.bbi.2015.01.001.
29. Shaik JSB, Poloyac SM, Kochanek PM, Alexander H, Tudorascu DL, Clark RSB, et al. 20-Hydroxyeicosatetraenoic Acid Inhibition by HET0016 Offers Neuroprotection, Decreases Edema, and Increases Cortical Cerebral Blood Flow in a Pediatric Asphyxial Cardiac Arrest Model in Rats. *Journal of Cerebral Blood Flow and Metabolism* 2015; 35(11):1757-1763. http://dx.doi.org/10.1038/jcbfm.2015.117.
30. Kasuga K, Yang R, Porter TF, Agrawal N, Petasis NA, Irimia D, et al. Rapid appearance of resolvin precursors in inflammatory exudates: novel mechanisms in resolution. *J Immunol* 2008; 181(12):8677-8687. 10.4049/jimmunol.181.12.8677.
31. Zuo G, Zhang D, Mu R, Shen H, Li X, Wang Z, et al. Resolvin D2 protects against cerebral ischemia/reperfusion injury in rats. *Mol Brain* 2018; 11(1):9. 10.1186/s13041-018-0351-1.
32. Bisicchia E, Sasso V, Catanzaro G, Leuti A, Besharat ZM, Chiacchiarini M, et al. Resolvin D1 Halts Remote Neuroinflammation and Improves Functional Recovery after Focal Brain Damage Via ALX/FPR2 Receptor-Regulated MicroRNAs. *Mol Neurobiol* 2018; 55(8):6894-6905. 10.1007/s12035-018-0889-z.
33. Belayev L, Mukherjee PK, Balaszczuk V, Calandria JM, Obenaus A, Khoutorova L, et al. Neuroprotectin D1 upregulates Iduna expression and provides protection in cellular uncompensated oxidative stress and in experimental ischemic stroke. *Cell Death Differ* 2017; 24(6):1091-1099. 10.1038/cdd.2017.55.
34. Gross GJ, Hsu A, Falck JR, Nithipatikom K. Mechanisms by which epoxyeicosatrienoic acids (EETs) elicit cardioprotection in rat hearts. *J Mol Cell Cardiol* 2007; 42(3):687-691. 10.1016/j.yjmcc.2006.11.020.
35. Motoki A, Merkel MJ, Packwood WH, Cao Z, Liu L, Iliff J, et al. Soluble epoxide hydrolase inhibition and gene deletion are protective against myocardial ischemia-reperfusion injury in vivo. *Am J Physiol Heart Circ Physiol* 2008; 295(5):H2128-2134. 10.1152/ajpheart.00428.2008.
36. Kain V, Liu F, Kozlovskaya V, Ingle KA, Bolisetty S, Agarwal A, et al. Resolution Agonist 15-epi-Lipoxin A4 Programs Early Activation of Resolving Phase in Post-Myocardial Infarction Healing. *Sci Rep* 2017; 7(1):9999. 10.1038/s41598-017-10441-8.
37. Halade GV, Kain V, Serhan CN. Immune responsive resolvin D1 programs myocardial infarction-induced cardiorenal syndrome in heart failure. *FASEB J* 2018; 32(7):3717-3729. 10.1096/fj.201701173RR.
38. Kain V, Ingle KA, Colas RA, Dalli J, Prabhu SD, Serhan CN, et al. Resolvin D1 activates the inflammation resolving response at splenic and ventricular site following myocardial infarction leading to improved ventricular function. *Journal of Molecular and Cellular Cardiology* 2015; 84:24-35. 10.1016/j.yjmcc.2015.04.003.

39. Keyes KT, Ye Y, Lin Y, Zhang C, Perez-Polo JR, Gjorstrup P, et al. Resolvin E1 protects the rat heart against reperfusion injury. *Am J Physiol Heart Circ Physiol* 2010; 299(1):H153-164. 10.1152/ajpheart.01057.2009.
40. Chen J, Shetty S, Zhang P, Gao R, Hu Y, Wang S, et al. Aspirin-triggered resolvin D1 down-regulates inflammatory responses and protects against endotoxin-induced acute kidney injury. *Toxicology and applied pharmacology* 2014; 277(2):118-123. 10.1016/j.taap.2014.03.017.
41. Zhao Yl Fau - Zhang L, Zhang L Fau - Yang Y-Y, Yang Yy Fau - Tang Y, Tang Y Fau - Zhou J-J, Zhou Jj Fau - Feng Y-Y, Feng Yy Fau - Cui T-L, et al. Resolvin D1 Protects Lipopolysaccharide-induced Acute Kidney Injury by Down-regulating Nuclear Factor-kappa B Signal and Inhibiting Apoptosis. (2542-5641 (Electronic)).
42. Duffield JS, Hong S, Vaidya VS, Lu Y, Fredman G, Serhan CN, et al. Resolvin D series and protectin D1 mitigate acute kidney injury. *J Immunol* 2006; 177(9):5902-5911. http://www.jimmunol.org/content/jimmunol/177/9/5902.full.pdf.
